# The Lysophospholipase PNPLA7 Controls Hepatic Choline and Methionine Metabolism

**DOI:** 10.3390/biom13030471

**Published:** 2023-03-03

**Authors:** Sayaka Harada, Yoshitaka Taketomi, Toshiki Aiba, Mai Kawaguchi, Tetsuya Hirabayashi, Baasanjav Uranbileg, Makoto Kurano, Yutaka Yatomi, Makoto Murakami

**Affiliations:** 1 Laboratory of Microenvironmental and Metabolic Health Sciences, Center for Disease Biology and Integrative Medicine, Graduate School of Medicine, The University of Tokyo, Tokyo 113-8655, Japan; 2 Department of Radiation Effects Research, National Institutes for Quantum and Radiological Science and Technology, Chiba 263-8555, Japan; 3 Laboratory of Biomembrane, Department of Basic Medical Sciences, Tokyo Metropolitan Institute of Medical Science, Tokyo 156-8506, Japan; 4 Department of Clinical Laboratory Medicine, The University of Tokyo, Tokyo 113-8655, Japan

**Keywords:** lysophospholipase, phospholipase A_2_, phosphatidylcholine, lysophosphatidylcholine, glycerophosphocholine, choline, methionine, S-adenosylmethionine, methyl group, liver

## Abstract

The in vivo roles of lysophospholipase, which cleaves a fatty acyl ester of lysophospholipid, remained unclear. Recently, we have unraveled a previously unrecognized physiological role of the lysophospholipase PNPLA7, a member of the Ca^2+^-independent phospholipase A_2_ (iPLA_2_) family, as a key regulator of the production of glycerophosphocholine (GPC), a precursor of endogenous choline, whose methyl groups are preferentially fluxed into the methionine cycle in the liver. PNPLA7 deficiency in mice markedly decreases hepatic GPC, choline, and several metabolites related to choline/methionine metabolism, leading to various symptoms reminiscent of methionine shortage. Overall metabolic alterations in the liver of *Pnpla7*-null mice in vivo largely recapitulate those in methionine-deprived hepatocytes in vitro. Reduction of the methyl donor *S*-adenosylmethionine (SAM) after methionine deprivation decreases the methylation of the *PNPLA7* gene promoter, relieves PNPLA7 expression, and thereby increases GPC and choline levels, likely as a compensatory adaptation. In line with the view that SAM prevents the development of liver cancer, the expression of PNPLA7, as well as several enzymes in the choline/methionine metabolism, is reduced in human hepatocellular carcinoma. These findings uncover an unexplored role of a lysophospholipase in hepatic phospholipid catabolism coupled with choline/methionine metabolism.

## 1. Introduction

The phospholipase A_2_ (PLA_2_) superfamily comprises a group of lipolytic enzymes that typically hydrolyze the *sn*-2 position of glycerophospholipids (phospholipids hereafter) to release fatty acids and lysophospholipids. The mammalian genome encodes more than 50 PLA_2_s or related enzymes, which are classified into several subfamilies on the basis of their structural and functional properties, including secreted PLA_2_s (sPLA_2_s), cytosolic PLA_2_s (cPLA_2_s), Ca^2+^-independent PLA_2_s (iPLA_2_s, also called patatin-like phospholipases (PNPLAs)), platelet-activating factor acetylhydrolases (PAF-AHs), lysosomal PLA_2_ (LPLA_2_), PLA/acyltransferases (PLAATs), α/β hydrolases (ABHDs), and so on [1]. In general, the PLA_2_ family has long been implicated in signal transduction by producing lipid mediators derived from polyunsaturated fatty acids (arachidonic acid in particular) and lysophospholipids. However, this principal concept is not sufficient for fully explaining the diverse biological roles of PLA_2_s, since individual PLA_2_s display distinct substrate specificities and some of these enzymes catalyze even non-PLA_2_ reactions, such as phospholipase A_1_ (PLA_1_), neutral lipid lipase and transacylase reactions, rendering the understanding of PLA_2_-driven lipid metabolism to be more complex than previously thought. It has long been considered that the cellular levels of lysophospholipids, a primary PLA_2_ reaction product, must be tightly controlled through reacylation by acyltransferases or degradation by lysophospholipases, since their unusual accumulation can lead to uncontrolled signaling due to their pleiotropic actions as a class of lipid mediators [2] and can also disturb membrane integrity and even cause cell lysis due to their detergent-like nature [3]. Although several PLA_2_ enzymes exhibit lysophospholipase activity at least in vitro, the biological importance of this enzymatic reaction in vivo remained largely unclear.

**Figure 1:**
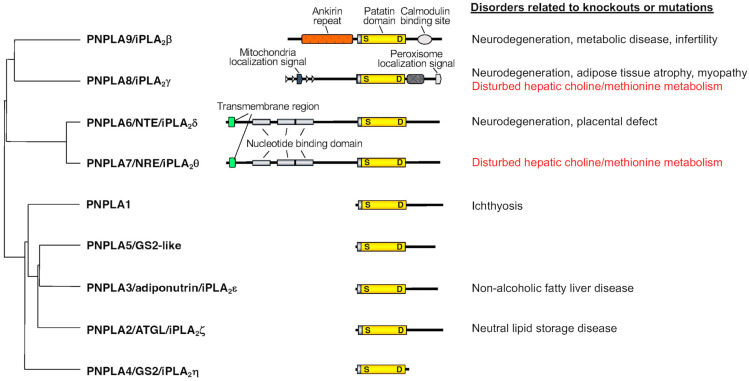
The PNPLA/iPLA_2_ family. Structural relationship of PNPLA enzymes (PNPLA1~9), as well as representative disorders caused by knockouts or mutations of these enzymes in mice and humans, are indicated. In principle, PNPLA1~5 act on neutral lipids, while PNPLA6~9 act on phospholipids. The functional association of PNPLA7 and PNPLA8 with hepatic choline/methionine metabolism, on which we put a specific focus in this review, is highlighted in red.

The PNPLA/iPLA_2_ family contains nine enzymes in humans, which share a patatin domain that was initially discovered in patatin (iPLA_2_α), a potato protein [1]. In general, enzymes with a large and unique N-terminal region (PNPLA6~9) act on phospholipids, while those lacking the N-terminal domain (PNPLA1~5) act on neutral lipids (Figure 1). The PNPLA/iPLA_2_ family is widely conserved in eukaryotes including yeast, ameba, plants, nematodes, flies, fish, mice and humans, suggesting that the enzymes in this family regulate various forms of homeostatic lipid metabolism that are fundamental for life. Indeed, genetic deletions or mutations of these enzymes cause various and often severe symptoms related to metabolic and neurological dysfunctions. The properties and regulatory functions of several PNPLA enzymes (PNPLA1, 2, 3, 6, 8 and 9) and their association with human diseases have been well documented in recent reviews [1,4,5,6,7]. Herein, we put a specific focus on the biological function of PNPLA7, one of the least characterized members of the PNPLA family, in phospholipid catabolism as a “lysophospholipase”.

## 2. Properties of PNPLA7, the Closest Paralog of PNPLA6

PNPLA6, also known as neuropathy target esterase (NTE) or iPLA_2_δ, was originally identified in 1998 as a key factor responsible for organophosphate-induced delayed neuropathy (OPIDN), a neurodegenerative disorder that occurs within a few weeks after exposure to pesticides and nerve agents containing organophosphates [8]. PNPLA6 displays phospholipase B (PLA_1_/PLA_2_ + lysophospholipase) activity, hydrolyzing both the *sn*-1 and *sn*-2 fatty acyl chains of phosphatidylcholine (PC) and lysophosphatidylcholine (LPC) [9,10,11]. Mutations in the human *PNPLA6* gene are linked to rare inherited diseases termed PNPLA6-related disorders, with clinical symptoms including spastic paraplegia, ataxia, hypogonadism, and chorioretinal dystrophy, reminiscent of the OPIDN pathology [12].

PNPLA7, also referred to as NTE-related esterase (NRE) or iPLA_2_θ, was identified in 2008 as the closest paralog of PNPLA6 [13]. The amino acid sequences of PNPLA6 and PNPLA7 are ~60% identical, with the N-terminal side facing the endoplasmic reticulum (ER) across the transmembrane domain and the C-terminal side including the catalytic patatin domain facing the cytosol (Figure 1). PNPLA7 displays lysophospholipase activity, hydrolyzing the *sn*-1 or *sn*-2 ester of LPC, although it does not catalyze the hydrolysis of PC via PLA_1_/PLA_2_ activity [13,14]. Both PNPLA6 and PNPLA7 are widely expressed in various tissues, with PNPLA6 being abundantly expressed in the central nervous system and PNPLA7 in metabolically active tissues including skeletal muscle, liver, and adipose tissue [14]. Expression of PNPLA7, but not PNPLA6, is upregulated in the liver and adipose tissue during fasting, suggesting its nutritional regulation [14]. In 3T3-L1 adipocytes, expression of PNPLA7 is downregulated by insulin [13]. Upon elevated fatty acid flux, the catalytic domain of PNPLA7 targets cellular lipid droplets in response to increased cAMP levels [15]. A recent study has reported that knockdown of hepatic PNPLA7 using adenoviral shRNA delivery impairs the secretion of very low-density lipoprotein (VLDL), proposing that PNPLA7 stabilizes ApoE through protein–protein interaction independent of its lysophospholipase activity [16]. However, biological importance of the lysophospholipase activity of PNPLA7 in vivo remains unclear.

**Figure 2:**
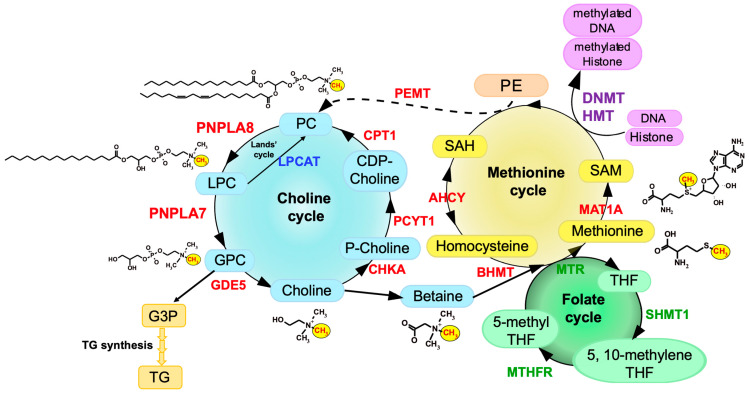
An overview of hepatic choline/methionine metabolism. Key metabolites and enzymes in this metabolic pathway are highlighted. Two PNPLA enzymes, PNPLA8 and PNPLA7, contribute sequentially to the mobilization of choline from membrane PC. Methyl groups (CH_3_) of choline are fluxed into the methionine cycle via betaine. The methionine cycle is coupled with histone and DNA methylation, PC biosynthesis via PEMT, and the folate cycle as another source of methyl group. The PNPLA8/PNPLA7-driven choline is also utilized for PC regeneration through the CDP-choline (Kennedy) pathway, constituting the choline cycle. Chemical structures of PC, LPC, GPC, choline, betaine, methionine and SAM are shown. For details, please see the text. SHMT1, serine hydroxymethyltransferase 1; MTHFR, methylenetetrahydrofolate reductase.

In our ongoing efforts to clarify the biological roles of the PLA_2_ family by gene targeting [17,18,19,20], we have recently uncovered a previously unrecognized role of PNPLA7 as a bona fide lysophospholipase in the liver [14]. The main physiological role of the lysophospholipase PNPLA7 is not merely to remove otherwise toxic LPC when accumulated in excess or to modulate lysophospholipid mediator signaling, but rather to generate glycerophosphocholine (GPC), a hydrolytic product of LPC, as a precursor of free choline, whose labile methyl groups are then preferentially fluxed into the methionine cycle (Figure 2). Impairment of this homeostatic process by PNPLA7 deficiency results in striking phenotypes reminiscent of impaired choline/methionine metabolism, as described below.

## 3. Choline/Methionine Metabolism

Choline, a quaternary amine having three methyl groups, is an essential dietary nutrient for normal body function due to its broad range of functions as a precursor of the neurotransmitter acetylcholine, as a structural component of membrane PC and sphingomyelin, and as a source of methyl group metabolism [21]. Since the amount of choline generated endogenously is thought to be insufficient to meet its demand, it needs to be obtained from food. The majority of cellular choline exists as PC in cellular membranes, indicating that this phospholipid represents the most abundant reserve pool of total cellular choline. PC can be produced from choline through de novo synthesis called the CDP-choline pathway or Kennedy pathway, in which choline is sequentially converted to phosphocholine by choline kinase (CHKA or CHKB), to CDP-choline by phosphocholine cytidylyltransferase (PCYT1), and then to PC by choline phosphotransferase (CPT1). Liver has an alternative PC-biosynthetic route that involves three-step methylation of phosphatidylethanolamine (PE) to generate PC by PE *N*-methyltransferase (PEMT), which accounts for 5–40% of total hepatic PC synthesis and is the only known route for de novo biosynthesis of the choline moiety in mammals [22]. PC is hydrolyzed by PLA_1_ or PLA_2_ to LPC, which is converted back to PC by lysophospholipid acyltransferases, a pathway known as the Lands’ cycle [23,24]. Additionally, LPC is further hydrolyzed by lysophospholipase to give rise to GPC, which is further split into choline and glycerol-3-phosphate (G3P) by glycerophosphodiester phosphodiesterase (GDE) [25]. The choline generated thus far is utilized for the regeneration of PC, constituting the choline cycle. In addition, methyl groups of choline are utilized for the biosynthesis of methionine specifically in hepatocytes.

Methionine is an essential and sulfur-containing amino acid that participates in various biological events, such as methylation of DNA and histones for epigenetic regulation of gene expression, redox maintenance, polyamine generation, and protein synthesis, and is also coupled with one-carbon metabolism through the folate cycle [26]. In the methionine cycle, homocysteine (Hcy) is metabolized to methionine by either methionine synthase (MTR), which transfers a methyl group from 5-methyl tetrahydrofolate (THF) in the folate cycle in concert with vitamin B_12_, or betaine homocysteine S-methyltransferase (BHMT), which transfers a methyl group from betaine [27,28]. Since betaine is an oxidized metabolite of choline, choline and methionine are interconnected via betaine by choline oxidase and BHMT. Dietary methionine is partitioned and, when not incorporated into proteins, is adenylated to give rise to S-adenosylmethionine (SAM) by methionine adenosyltransferase 1A (MAT1A) [29]. SAM is required for transmethylation reactions for nucleic acids, proteins, lipids, and small molecules [30,31]. In mammals, ~50% of methionine metabolism and ~85% of transmethylation occur in the liver. SAM is converted to S-adenosylhomocysteine (SAH), a byproduct of transmethylation reactions. SAH is hydrolyzed by SAH hydrolase (AHCY) to adenosine and Hcy, the latter of which is then converted back to methionine by receiving a methyl group from 5-methyl-THF/vitamin B_12_ via the folate cycle or from betaine via the choline cycle [32]. Cellular levels of SAM vary depending on cellular nutrient conditions and affect the activity of histone methyltransferases (HMTs) and DNA methyltransferases (DNMTs) [33,34]. Histone methylation by HMTs is crucial for the regulation of chromatin remodeling and gene transcription. Methylation of the CpG islands in gene promoters by DNMTs is an important component for the epigenetic code, and many genes become abnormally methylated during tumorigenesis [35,36]. The methionine cycle is also linked to other metabolic pathways such as polyamine synthesis and transsulfuration [33,37]. Furthermore, the methyl group of SAM is transferred by PEMT to PE, resulting in the generation of PC, as mentioned above. An overview of the connection of the methionine cycle with the choline cycle is illustrated in Figure 2.

The importance of choline/methionine metabolism has been demonstrated by genetic or dietary models that harbor chronic choline/methionine deficiency or excess in rodents. Mice fed a choline/methionine-deficient diet exhibit various metabolic abnormalities including decreased PC synthesis, inadequate supply of labile methyl groups, and adaptive changes in lipid metabolism, which lead to hepatic steatosis, impaired VLDL secretion, muscle weakness, reduced adiposity, hypoglycemia, hypermetabolism, and growth retardation [38,39,40]. Moreover, gene knockout of BHMT, a rate-limiting enzyme for the methyl group flux from choline to methionine via betaine, mimics these phenotypes [27]. However, the PC-catabolic pathway that supplies endogenous choline (PC → LPC → GPC → choline) has currently received less attention, and accordingly, molecular entity of the enzyme(s) responsible for this process and its biological importance remain unknown.

We have recently demonstrated that PNPLA7, by acting as a lysophospholipase, is responsible for the conversion of LPC to GPC in this metabolic pathway to promote the generation of free choline, whose methyl groups are then transferred into the methionine cycle (Figure 2). PNPLA7 deficiency markedly decreases the mobilization of GPC and choline from membrane PC in the liver, leading to the phenotypes reminiscent of methionine insufficiency. Moreover, striking phenotypic similarity between *Pnpla7*^−/−^ and *Pnpla8*^−/−^ mice has revealed that PNPLA8 (also known as group VIB PLA_2_ or iPLA_2_γ), by regulating the conversion of PC to LPC as a PLA_1_/PLA_2_, lies upstream of PNPLA7 in the hepatic PC-catabolic pathway to mobilize endogenous choline [14] (Figure 2).

## 4. Phenotypes of *Pnpla7*-Deficient Mice

*Pnpla7*^−/−^ mice are born normally, yet they suffer from growth retardation after weaning and die within a few months [14]. *Pnpla7*^−/−^ mice exhibit kyphosis (excessive convex backward curvature of the spine) with muscle weakness and have markedly reduced visceral and subcutaneous fats, in which adipocytes contain smaller lipid droplets and more mitochondria and show some features of beige adipocytes with greater expression of thermogenic markers such as UCP1. In addition, *Pnpla7*^−/−^ mice display increases in food intake, locomotion, oxygen consumption, and dependence on lipid over carbohydrate as an energy source. The serum levels of glucose, TG, insulin and leptin are decreased, while the level of the ketone body β-hydroxybutyrate, a marker of starvation, is elevated, in *Pnpla7*^−/−^ mice. Although insulin sensitivity is unaltered, hepatic gluconeogenesis and VLDL secretion are compromised by PNPLA7 deficiency. Probably due to the lower levels of blood glucose and TG as fuels, *Pnpla7*^−/−^ mice have a lower body temperature. Hepatic expression of genes related to lipogenesis is decreased, while that of genes related to lipolysis and fatty acid β-oxidation is elevated, in *Pnpla7*^−/−^ mice. Furthermore, PNPLA7 deficiency markedly increases the blood level of the hepatokine FGF21, which could mediate some of the metabolic phenotypes described above. Importantly, the increased FGF21 expression in *Pnpla7*^−/−^ hepatocytes is reversed to its normal level by methionine (rather than choline) supplementation, implying that methionine is a key metabolite acting downstream of PNPLA7.

Metabolome analysis has revealed that the levels of GPC, choline and phosphocholine are markedly reduced, while those of LPC species are reciprocally elevated, in the liver of *Pnpla7*^−/−^ mice relative to *Pnpla7*^+/+^ mice, indicating that PNPLA7 acts as a major lysophospholipase that hydrolyzes LPC to GPC in this tissue [14]. In corroboration, acute knockdown of PNPLA7 in cultured hepatocytes decreases, whereas its overexpression conversely increases, cellular GPC levels. In contrast, PNPLA7 deficiency does not profoundly affect GPC levels in non-hepatic tissues such as adipose tissue, brain and kidney, where PNPLA6 (or some other enzymes) may play a major or redundant role in GPC production. Notably, hepatic levels of a series of metabolites related to the methionine cycle such as betaine, methionine, and SAM, as well as the ratio of SAM/SAH (an indicator of the methylation flux), are markedly reduced in *Pnpla7*^−/−^ mice. These metabolic changes are accompanied by compensatory upregulation of biosynthetic enzymes for the methionine-related metabolites including *Bhmt, Mat1a* and *Ahcy,* in line with their upregulation in other models of methionine insufficiency [41]. Since the methionine cycle is tightly linked to the transsulfuration and polyamine pathways [42], hepatic levels of several metabolites in these metabolic pathways are also altered in *Pnpla7*^−/−^ liver. These results suggest that PNPLA7 deficiency reduces the flux of labile methyl groups from choline driven by the PC-catabolic pathway to the methionine cycle, in association with marked changes in the levels of related metabolites in the liver, thus underscoring a crucial role of this lysophospholipase in the mobilization of hydrophilic choline from hydrophobic PC. Indeed, dietary methionine restriction leads to reduced body weight, increased energy consumption, adipose tissue atrophy, hypoglycemia, hypolipidemia, hypothermia, hypoinsulinemia with normal insulin sensitivity, and increased FGF21 expression [40], all of which are recapitulated in *Pnpla7*^−/−^ mice.

SAM serves as an essential methyl donor for methylation of histones and DNA, thereby epigenetically regulating the expression of various genes [33,37]. Notably, PNPLA7-driven SAM production is also coupled with the methylation of histones and gene promoters that could affect gene expression [14]. As for histone methylation, PNPLA7 deficiency reduces the methylation of H3:K79, which is regulated rather specifically by Dot1L, an HMT whose activity is more sensitive to subtle changes in the concentration of SAM than that of other HMTs [43], accounting for the selective reduction of H3:K79 methylation in the liver of *Pnpla7*^−/−^ mice. Since Dot1L-mediated H3:K79 methylation generally enhances the transcription of target genes [44], this histone modification may be involved in the expression of some genes that are decreased in *Pnpla7*^−/−^ mice. Furthermore, the promoter regions of several genes potentially associated with hepatic fibrosis, signal transduction, redox regulation, and oncogenic transformation have lower methylation levels in *Pnpla7*^−/−^ liver, suggesting that the reduced SAM flux by PNPLA7 deficiency also decreases DNA methylation by DNMTs. Although the precise association of these histone/DNA methylations with various phenotypes in *Pnpla7^−^*^/−^ mice needs further elucidation, this finding provides, to the best of our knowledge, the first evidence that PC/LPC catabolism regulated by a particular member of the PLA_2_ family is linked to epigenetic regulation of gene expression.

## 5. Enzymes That Lie Upstream and Downstream of PNPLA7

Knowing that PNPLA7 is responsible for the conversion of LPC to GPC in the hepatic PC-catabolic pathway, which enzymes lie upstream (PC → LPC) and downstream (GPC → choline) of PNPLA7 in this tissue? Notably, of the knockout mouse strains for various PLA_2_s examined so far [1], only *Pnpla8*^−/−^ mice share several common features with *Pnpla7*^−/−^ mice. These phenotypes include reduced body weight and adiposity, muscle weakness leading to kyphosis, increased oxygen consumption, hypothermia, and reduced blood levels of glucose, TG, insulin, and leptin [45,46]. Among the PLA_2_ family, PNPLA8 shows the highest expression in mouse liver, with the magnitude order of PNPLA8 > PNPLA9 = PNPLA7 > PNPLA6 >> other PLA_2_s. Importantly, as in *Pnpla7*^−/−^ mice, hepatic levels of GPC, choline, betaine, and SAM are substantially reduced in *Pnpla8*^−/−^ mice [14]. Moreover, more than one third of the upregulated or downregulated genes, including those related to choline/methionine metabolism, fat synthesis and utilization, and glucose metabolism, in *Pnpla8*^−/−^ liver are commonly changed in *Pnpla7*^−/−^ liver. Hepatic expression of FGF21 is increased in both *Pnpla7*^−/−^ and *Pnpla8*^−/−^ mice. Thus, the phenotypes observed in *Pnpla8*^−/−^ mice are strikingly similar to (even though milder than) those observed in *Pnpla7*^−/−^ mice, implying that PNPLA7 and PNPLA8 lie in the common metabolic pathway. Considering that PNPLA8 displays PLA_1_/PLA_2_ rather than lysophospholipase activity [47], it is likely that PNPLA8 is a major upstream PLA_1_/PLA_2_ involved in the conversion of PC to LPC in the context of hepatic PC catabolism. Although PNPLA8 has currently been implicated in membrane remodeling and lipid meditator generation [45,46,47,48,49], this study has provided another insight into the role of this iPLA_2_ isoform in hepatic choline/methionine metabolism.

Following the lysophospholipase reaction, GPC is degraded into choline and G3P by GDE enzymes. Of the seven GDE enzymes identified in mammals, only GDE5/GPCPD1 is markedly upregulated in the livers of both *Pnpla7*^−/−^ and *Pnpla8^−^*^/−^ mice [14]. This upregulation can be regarded as a compensatory adaptation and implies that this GDE isoform is mainly responsible for the conversion of GPC to choline downstream of PNPLA7, although this point should be confirmed using *Gde5/Gpcpd1*-deficient mice in a future study. G3P, another product of the GDE reaction, is a precursor of TG biosynthesis. In *Pnpla7*^−/−^ liver, the metabolic flow from G3P to TG is also mitigated, resulting in reduced hepatic fat accumulation. This agrees with the view that a large fraction of the glycerol backbone of TG is derived from that of PC in hepatocytes [50]. The overall picture of the hepatic PC-catabolic pathway involving PNPLA8, PNPLA7 and GDE5/GPCPD1, which is coupled with the methionine cycle, is summarized in Figure 2.

## 6. Regulation of PNPLA7 Expression in Hepatocytes by Methionine Availability

**Figure 3:**
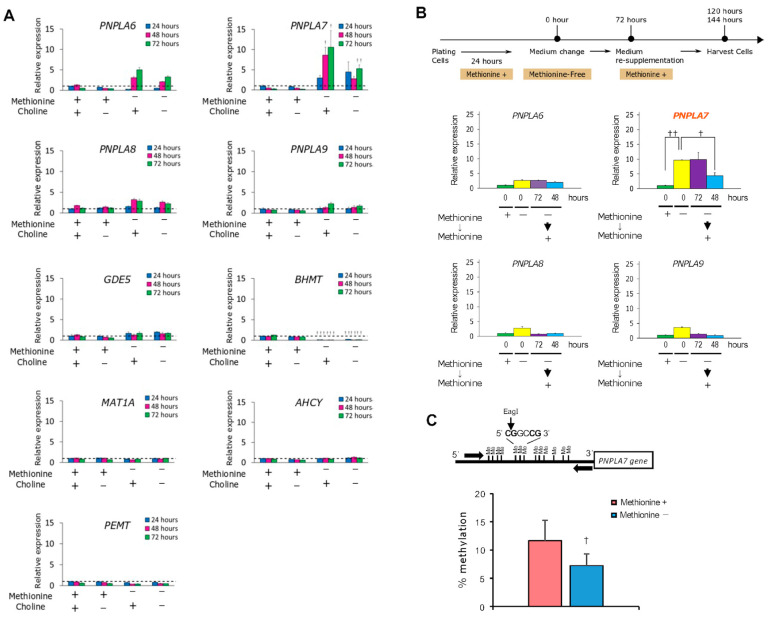
Methionine deprivation upregulates *PNPLA7* expression in HepG2 cells by reducing the methylation level in the *PNPLA7* promoter. (**A**) Quantitative RT-PCR (qPCR) of PNPLAs and enzymes related to choline/methionine metabolism in cells cultured for the indicated times in the presence (+) or absence (−) of choline and/or methionine, with the expression level of each gene under normal culture condition as 1 (dashed lines). Values are mean ± SEM (*n* = 6); †, *p* < 0.05; ††, *p* < 0.01 versus cells cultured with methionine and choline for 24 h; two-tailed Student’s *t*-test. (**B**) HepG2 cells were cultured for 72 h in methionine-free medium and then cultured for the indicated times in medium supplemented with methionine (**Top panel**). Graphs show qPCR of PNPLA enzymes upon methionine deprivation followed by its re-supplementation, with the expression level of each gene in cells cultured with methionine for 72 h as 1. Values are mean ± SEM (*n* = 3); ††, *P* < 0.01; ANOVA with Turkey post hoc test. (**C**) Methylation analysis of the *PNPLA7* promoter by MSRE-PCR. An image of the methylation status in the *PNPLA7* promoter in HepG2 cells and the cleavage site of *Eag*I, and PCR primers (arrows) used in the MSRE-PCR analysis are shown (**Top panel**). A graph shows frequency of the methylated *Eag*I-CpG site in the *PNPLA7* promoter in HepG2 cells cultured with or without methionine. Values are mean ± SEM (*n* = 4); †, *p* < 0.05; two-tailed Student’s *t*-test. In the qPCR analysis (**A**,**B**), total RNA was extracted from the cells using the TRIzol Reagent (Thermo Fischer Scientific, Waltham, MA, USA, #15596018), cDNA was synthesized using random primers and a High-Capacity cDNA Reverse-Transcription Kit (Thermo Fischer Scientific, #4368813), and PCR was carried out using TaqMan Gene Expression Master Mix (Thermo Fischer Scientific, #4369016) and TaqMan Gene Expression Assays (TaqMan probe-primer sets) on a StepOnePlus Real-Time PCR System (Thermo Fischer Scientific). The probe-primer sets used were as follows: *PNPLA6*, Hs00210447_m1; *PNPLA7*, Hs00173472_m1; *PNPLA8*, Hs00358567_m1; *PNPLA9/PLA2G6*, Hs00913513_m1; *GDE5/GPCPD1*, Hs00325631_m1; *AHCY*, Hs00426322_m1; *BHMT,* Hs01566156_m1; *MAT1A,* Hs01547962_m1; and *PEMT*, Hs01002999_m1. Expression levels of these genes were normalized to *GAPDH* (4326317E, Thermo Fischer Scientific). In the MSRE-PCR analysis [52,53] (**C**), genomic DNA was digested with the methylation-sensitive restriction enzyme *Eag*I. The digested DNA samples were subjected to qPCR by a LightCycler (Roche Diagnostics GmbH) with a forward primer 5′-GGTTCGTGCAGATCAAGGAG-3′ and a reverse primer 5′-GTTGTCAGGGTCGAAGGTACC-3′. The ratio of target DNA amounts was determined using the ΔΔCt method in which the PCR efficiency was considered 2 and the methylation levels (expressed as % methylation) at the *Eag*I-CpG site were calculated relative to those of untreated DNA, whose methylation level was set at 100%.

Although *Pnpla7* expression is upregulated in the liver and adipose tissue of mice after fasting [13,14], it remains unknown as regards to which nutritional component(s) could influence its expression. Since PNPLA7 is functionally linked to choline/methionine metabolism, we hypothesized that its expression would be regulated by some metabolites in this metabolic pathway. To test this, the human hepatoblastoma cell line HepG2 was cultured in medium supplemented with or without choline and/or methionine. We found that the expression of *PNPLA7,* and to a much lesser extent that of *PNPLA6, PNPLA8* and *PNPLA9,* was upregulated in HepG2 cells cultured without methionine. The increased expression of *PNPLA7* was observed in cells cultured for 24–72 h in methionine- or choline/methionine-depleted, but not choline-depleted, medium (Figure 3A). Other genes related to choline/methionine metabolism were barely affected (*GDE5, MAT1, AHCY* and *PEMT*) or decreased (*BHMT*) by methionine depletion. Methionine re-supplementation to the methionine-free culture resulted in downregulation of *PNPLA7* to a level comparable to that in the cells cultured in normal medium (Figure 3B). Thus, *PNPLA7* expression is controlled by availability of methionine rather than choline, being upregulated after methionine depletion and downregulated after methionine supplementation.

The promoter region of the *PNPLA7* gene is hypermethylated in several human hepatocellular carcinoma cell lines, including HepG2 and Huh7, and treatment of the cells with a DNMT inhibitor increases *PNPLA7* expression [51]. We therefore speculated that the reduction of cellular SAM levels by methionine withdrawal might decrease the methylation of the *PNPLA7* promoter, thus allowing upregulation of *PNPLA7* expression. To address this issue, we investigated the methylation level in the promoter region of the *PNPLA7* gene in HepG2 cells cultured with or without methionine by MSRE-PCR (methylation-sensitive restriction enzymes-polymerase chain reaction) analysis [52,53]. The cells cultured in methionine-free medium showed a lower methylation level at a CpG site in the *PNPLA7* promoter than those cultured in methionine-sufficient medium (Figure 3C). These results suggest that the decreased SAM flux in methionine-deprived cells results in reduced methylation of the *PNPLA7* promoter, thereby allowing increased *PNPLA7* expression. As described below, the increased *PNPLA7* expression in methionine-free culture is accompanied by increases in cellular GPC, choline, phosphocholine, and CDP-choline levels.

Although transcription factors responsible for the expression of *PNPLA7* remain to be determined, a database search of the ChIP-Atlas for transcription factors activated by fasting reveals that FOXO1 is a potential candidate that can bind to the promoter region of the *PNPLA7* gene. The transcriptional activity of FOXO1 is suppressed through phosphorylation by AKT downstream of the insulin signal, whereas it is activated through phosphorylation by JNK and AMPK under hypotrophic conditions when the insulin signal is attenuated [54]. In support of this idea, *Pnpla7* expression in mouse 3T3-L1 adipocytes is downregulated by insulin [13]. Whether FOXO1, or any other transcription factors, would be indeed involved in the regulation of PNPLA7 expression should await a future study.

## 7. Changes in Methionine-Related Metabolites in Hepatocytes Cultured in Methionine-Deprived Medium Are Similar to Those in the Liver of *Pnpla7*-Deficient Mice

**Figure 4:**
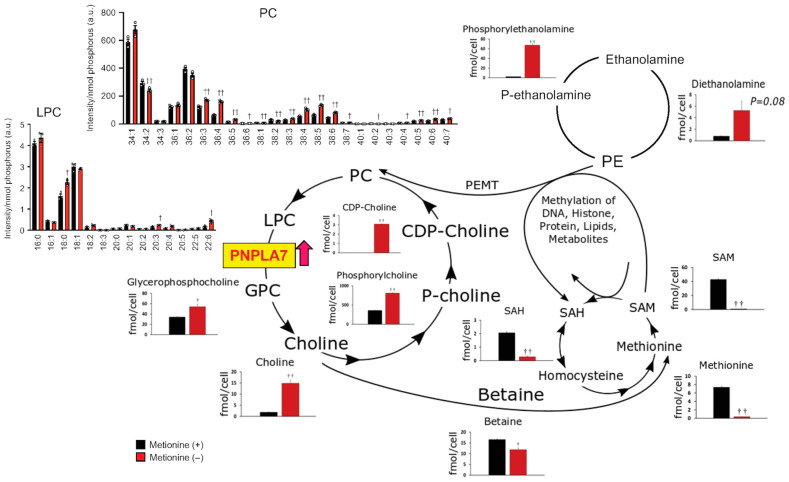
Effect of methionine deprivation on the choline and methionine cycles in HepG2 cells. Graphs represent the levels of individual metabolites in HepG2 cells cultured with (+) or without (−) methionine for 48 h. PNPLA7 is upregulated in cells cultured in methionine-free medium (see Figure 3). Quantification of lipids (PC, LPC, and PE) and water-soluble metabolites were performed by LC–MS and CE–MS, respectively [14]. Mean ± SEM (*n* = 4); †, *p* < 0.05; ††, *p* < 0.01; two-tailed Student’s *t*-test. For details, please see the text.

**Figure 5:**
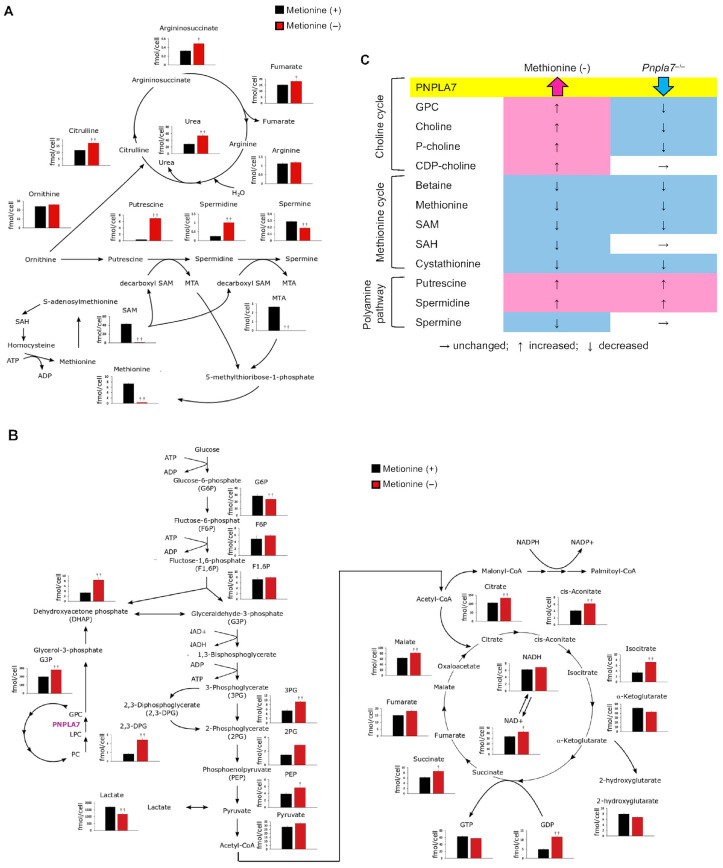
Effects of methionine deprivation on the urea cycle, polyamine synthesis, and methionine salvage pathway (**A**), and glycolysis and TCA cycle (**B**) in HepG2 cells. Graphs show the levels of individual metabolites in HepG2 cells cultured with (+) or without (−) methionine for 48 h. Quantification of each metabolite was performed by CE–MS. Mean ± SEM (*n* = 4); †, *p* < 0.05; ††, *p* < 0.01; two-tailed Student’s *t*-test. (**C**) Comparison of the changes in various metabolites in methionine-deprived HepG2 cells with those in the liver of *Pnpla7*^−/−^ mice [14]. In methionine-deprived HepG2 cells, the levels of metabolites in the choline cycle were increased because of the upregulation of PNPLA7, whereas in *Pnpla7*^−/−^ liver the absence of PNPLA7 decreased their levels. In both cases, the methionine flux decreased, resulting in similar alterations in multiple metabolites in the methionine cycle and the polyamine pathway. For details, please see the text.

To assess how methionine deficiency impacts cellular metabolism, we conducted global metabolome analysis of hydrophilic metabolites and hydrophobic phospholipids by liquid chromatography mass spectrometry (LC–MS) and capillary electrophoresis mass spectrometry (CE–MS), respectively, using HepG2 cells cultured in methionine-sufficient or methionine-depleted medium. The effect of methionine deficiency on HepG2 cells was confirmed by the cellular methionine level, which was dramatically reduced (Figure 4). SAM and SAH, which are immediate downstream metabolites of methionine, were also reduced markedly in methionine-depleted cells. SAM transfers its methyl group to PE to generate PC, a reaction catalyzed by PEMT [22,38,41], thus being coupled with the choline cycle. Interestingly, several metabolites in the choline cycle such as GPC, choline, phosphocholine, and CDP-choline were increased in methionine-depleted cells (Figure 4), suggesting that the choline cycle, involving PC catabolism and de novo synthesis (Kennedy pathway), is activated in response to the upregulation of PNPLA7 under the methionine-insufficient condition (Figure 3). Consistent with the downregulation of *BHMT* (Figure 3A), betaine was reduced in methionine-depleted cells (Figure 4), likely because choline is preferentially fluxed into the Kennedy pathway for de novo PC synthesis under the condition where the PEMT-driven PC synthesis is impeded [22,38,41]. Despite the reduced SAM flux and thereby reduced PEMT-dependent PC synthesis, the levels of PC and LPC species were unchanged or slightly increased (particularly those with polyunsaturated fatty acids) in methionine-deprived cells (Figure 4), further implying that PC biosynthesis was complemented by the Kennedy pathway. These results are consistent with the observations that, when the production of PC through PE methylation by PEMT is limited, the Kennedy pathway is complementarily activated to replenish PC, that the Kennedy pathway favors the biosynthesis of PC species with polyunsaturated fatty acids, and that an impairment of the PEMT pathway leads to upregulation of PC catabolism and choline recycling [22,38,41]. Furthermore, given that PNPLA7 also hydrolyzes lysophosphatidylethanolamine (LPE) [14,15], the increases of diethanolamine and phosphoethanolamine in methionine-depleted cells may reflect the fact that the upregulated PNPLA7 hydrolyzes LPE to produce these ethanolamine metabolites, which are likely utilized for regeneration of PE to maintain the levels of PE species (Figure 4). The amount of 5′-methylthioadenosine (MTA), one of the SAM derivatives in the methionine salvage pathway, was also markedly reduced in methionine-deficient cells (Figure 5A). MTA generation in the methionine salvage pathway is coupled with polyamine biosynthesis [55]. Indeed, the amounts of polyamines such as putrescine and spermidine were markedly elevated, while that of spermine was decreased, in methionine-deprived cells (Figure 5A), implying that the reduced metabolic flux from SAM to MTA eventually perturbed the polyamine pathway, leading to an accumulation of precursor polyamines (putrescine and spermidine) and a reduction of polyamine end-product (spermine). Putrescine is synthesized from ornithine, a metabolite utilized in the urea cycle. In correlation with the increase of precursor polyamines, several metabolites in the urea cycle, including citrulline, urea, argininosuccinate, and fumarate, were significantly elevated in methionine-depleted cells (Figure 5A).

Given that GPC is split into choline and G3P, the latter of which is utilized for glycolysis via dihydroxyacetone phosphate (DHAP), it was speculated that PNPLA7 upregulation in methionine-free culture would increase the G3P-driven glycolytic flux. Indeed, although the levels of glucose 6-phosphate (G6P), fructose 6-phosphate (F6P), and fructose 1,6-bisphosphate (F1,6BP) were unaffected, G3P and its downstream metabolites such as DHAP, glyceraldehyde 3-phosphate, phosphoenol pyruvate, and pyruvate were significantly increased in methionine-deprived cells (Figure 5B). Furthermore, in agreement with the increased glycolytic metabolites, various metabolites in the TCA cycle, such as citrate, cis-aconitate, isocitrate, succinate, fumarate, and malate, were increased in methionine-deficient cells (Figure 5B). An overall increase in the TCA cycle metabolites at the expense of a decrease in lactate suggests that methionine depletion increased aerobic respiration, which is consistent with the increased energy consumption in *Pnpla7*^−/−^ mice [14]. The increased NADPH/NADP^+^ ratio, as revealed by a slight increase in NADPH and a marked reduction in NADP^+^ after methionine depletion (Figure 5B), might reflect a decreased lipogenic flux, which depends on NADPH, in methionine-depleted cells, in line with the view that dietary methionine restriction [56] or *Pnpla7* deficiency [14] reduces hepatic lipogenesis in vivo.

Comparison of the changes in choline/methionine-related metabolites in methionine-deprived HepG2 cells and those in the liver of *Pnpla7*^−/−^ mice is summarized in Figure 5C. Overall changes in multiple methionine-related metabolites in methionine-deprived cells are largely even if not solely recapitulated in the liver of *Pnpla7*^−/−^ mice, providing further evidence that PNPLA7-driven phospholipid catabolism is functionally linked to choline/methionine metabolism.

## 8. PNPLA7 Expression Is Decreased in Human Hepatocellular Carcinoma (HCC)

**Figure 6:**
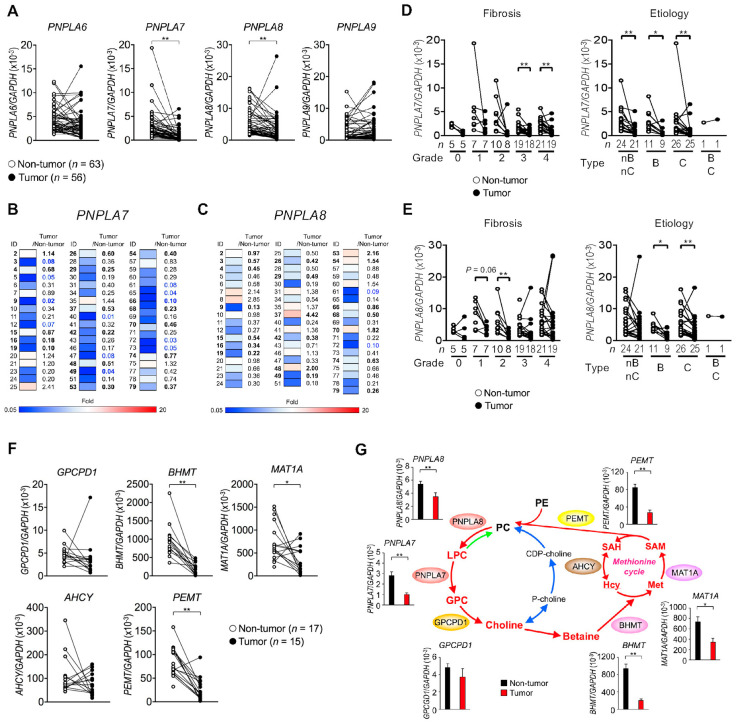
Reduced expression of *PNPLA7* and *PNPLA8* in human HCC. (**A**) qPCR analysis of *PNPLA6, PNPLA7, PNPLA8,* and *PNPLA9* in tumor and non-tumor tissues from human HCC patients. The expression was normalized to *GAPDH*. (**B**,**C**) Heatmap representations of *PNPLA7* (**B**) and *PNPLA8* (**C**) expression in individual patients. Sample IDs and expression ratios (tumor versus non-tumor; values of <0.1 are highlighted in blue) are indicated. Patients with non-B/non-C type HCC are shown in bold. (**D**,**E**) Expression of *PNPLA7* (**D**) and *PNPLA8* (**E**) in human HCC differing in the stage of fibrosis and etiology. In terms of etiology, nB/nC (non-B and non-C), B, C and B/C indicate HCC unrelated to HBV and HCV infection (probably associated with NAFLD and NASH), that are due to HBV infection, HCV infection, and both HBV and HCV infection, respectively. (**F**) Expression of enzymes related to choline/methionine metabolism in tumor and non-tumor tissues from human HCC patients. (**G**) Expression levels of key enzymes in tumor and non-tumor tissues from human HCC patients are summarized on the metabolic map of PC catabolism and the methionine cycle. Mean ± SEM, * *p* <0.05 and ** *p* < 0.01 versus non-tumor tissue. All of the enrolled patients underwent liver resection for treatment of HCC. Gender, age, and disease stages of the patients are summarized in Table S1.

Current evidence suggests that SAM deficiency favors HCC development, which can be prevented by an exogenous supply of SAM [57,58]. Indeed, *Mat1a*-deficient mice, which display hepatic SAM deficiency, are highly susceptible to liver cancer, whereas hepatoma cells transfected with *MAT1A* grow more slowly than control cells [32,59]. In human HCC (obtained from patients who had been treated in the Hepatobiliary Pancreatic Division, Department of Surgery, the University of Tokyo Hospital [60]) (Table S1), the expression levels of *PNPLA7* and *PNPLA8,* but not *PNPLA6* and *PNPLA9*, were significantly lower in tumor tissues than in non-tumor tissues (Figure 6A). Heatmap representation revealed that *PNPLA7* and to a lesser extent *PNPLA8* were markedly downregulated in tumor tissues relative to non-tumor tissues in most patients (Figure 6B,C), regardless of the stage or etiology of HCC (Figure 6D,E). The expression levels of enzymes in the methionine cycle (*BHMT, MAT1A,* and *PEMT*) were also lower in the tumors than in the controls (Figure 6F). Thus, multiple enzymes involved in PC catabolism and the methionine cycle were downregulated in HCC (Figure 6G), suggesting that the reduced methyl group flux through this metabolic pathway contributes to progression of HCC.

Since PNPLA7 expression is downregulated by insulin [13] and insulin signaling is constitutively activated in HCC [61], excess insulin signaling in liver cancer may lead to downregulation of PNPLA7 expression. As cancer stem cells exhibit a high dependence on exogenous methionine [62,63], high metabolic flux of exogenous methionine in association with SAM-dependent methylation at the initial stage of tumor development might ultimately lead to subsequent shutting down of the flux of endogenous methionine by downregulating key metabolic enzymes in the choline/methionine metabolism as a negative-feedback mechanism. In support of this idea, CpG hypermethylation in the *PNPLA7* and *MAT1A* promoters leads to downregulation of their expression in several human HCC cell lines [51,64]. Taken together, concomitant reduction of various enzymes involved in choline/methionine metabolism, particularly *PNPLA7* whose expression level is markedly reduced in most HCC patients, could be a novel diagnostic marker of this disease. Furthermore, control of the SAM level by manipulating the expression or activity of PNPLA7 might be a novel therapeutic strategy for the treatment of patients with HCC.

It should be noted, however, that the major drawback of the study design is its purely descriptive and correlative approach. Given the fact that there is virtually not a single functional study on human PNPLA7, such experiments would fill an important gap and strongly increase the impact of the study. Although knockdown of PNPLA7 decreases the cellular GPC level in mouse hepatocytes [14], its effect on the GPC level in human HepG2 cells is only marginal likely because the expression of PNPLA7, relative to that of its paralog PNPLA6, is repressed at a lower level due to its promoter methylation (see above) [51] and because PNPLA6 is compensatory upregulated following PNPLA7 knockdown (unpublished results) in this hepatoma cell line. Nevertheless, overexpression of PNPLA7 increases the GPC level in HepG2 cells [14], supporting the view that PNPLA7 is involved in GPC production in human hepatocytes as well. The functional redundancy or segregation between PNPLA6 and PNPLA7 in the regulatory production of GPC and downstream metabolites in various cells under various pathophysiological conditions is now under investigation.

## 9. Other Lysophospholipases

Beyond PNPLA7, several enzymes in the PLA_2_ family have been reported to possess lysophospholipase activity. As mentioned above, it is likely that PNPLA6 plays a compensatory or overlapping role with PNPLA7 in PC catabolism and recycling in tissue-specific contexts. Global *Pnpla6*-deficient (*Pnpla6*^−/−^) mice die in utero around E7.5 due to placental development [65]. Brain-specific *Pnpla6*-deficient mice display striking defects in motor coordination and neuronal degeneration with loss of Purkinje cells, disruption of the ER, accumulation of reticular aggregates, and an increase of PC [66], resembling the features of PNPLA6-related disorders [12] or hereditary spastic paraplegia [67] in humans. In these mice, degeneration of the distal parts of the longest spinal axons becomes evident, with massive axon swelling in both the ascending and descending sensory and motor spinal tracts [68], suggesting the PC/LPC catabolism driven by PNPLA6 in neurons may play a critical role in axonal membrane trafficking and secretion. All cPLA_2_ enzymes (group IVA-F PLA_2_s or cPLA_2_α-ζ) and PNPLA9 (group VIA PLA_2_ or iPLA_2_β) display lysophospholipase activity in vitro [69,70,71,72], yet the contribution of this activity to cPLA_2_- or iPLA_2_β-dependent biological processes remains unclear. Among the ABHD family, ABHD6, a monoacylglycerol lipase, also appears to function as a lysophospholipase and is involved in the regulation of endocannabinoid signaling and metabolic disease [73,74]. ABHD12 hydrolyzes lysophosphatidylserine (LysoPS) as a LysoPS lipase (LysoPS-specific lysophospholipase) and its genetic deletion or mutation increases LysoPS in the brain, leading to symptoms reminiscent of PHARC (polyneuropathy, hearing loss, ataxia, retinitis pigmentosa, and cataracts) [75]. ABHD12B deficiency also increases serum histamine levels due to the elevation of LysoPS, a bioactive lipid that augments mast cell activation [76]. Although LYPLA1 and LYPLA2 are referred to as lysophospholipases, it is now obvious that they act as acyl-thioesterases that catalyze the depalmitoylation of palmitoylated proteins [77].

## 10. Conclusions

Our study has opened new insights into the biological role of lysophospholipase in vivo, thus contributing to a conceptual advance in the research field of PLA_2_s and lipid metabolism. The PNPLA8/PNPLA7-driven PC catabolism is a missing piece of the puzzle that enables the recycling of choline and methyl groups between the choline cycle and the methionine cycle, which are interconnected by BHMT and PEMT in the liver. Since the information described here is based on the study using global *Pnpla7*^−/−^ mice, the possibility that some of the hepatic effects could be influenced by its deficiency in non-hepatic tissues such as brain and muscle should also be taken into consideration. Besides the role of PNPLA7 in choline/methionine metabolism, which is rather liver-specific, the broad distribution of PNPLA7 in many tissues that do not express BHMT implies that this lysophospholipase may also participate in other choline-related processes, which include not only recycling of choline into membrane PC through the Kennedy pathway, but also biosynthesis of sphingomyelin, generation of the neurotransmitter acetylcholine, activation of the sigma receptor that enhances Ca^2+^ signaling, and supply of GPC as an osmolyte, among others. A recent study has shown that PNPLA7 is abundantly expressed in macrophages and prevents their M1 polarization, possibly through sequestering pro-inflammatory LPC signaling [78]. It is also important to clarify the functional segregation or redundancy of the two related enzymes PNPLA6 and PNPLA7 in tissue-specific contexts. Lastly, beyond the inverse correlation between *PNPLA7* expression and human HCC, it remains obscure whether the present findings can be translated into human pathophysiology such as metabolic and neuronal diseases. Considering that mutations in the human *PNPLA6*, *PNPLA8,* or *PNPLA9* are linked to neurodegenerative diseases [1,4,5,11], it is tempting to speculate that disturbed phospholipid catabolism and turnover would commonly underly the neurodegenerative phenotypes caused by loss of this enzyme family in both mice and humans.

## Data Availability

The data that support the findings of this study are available from the corresponding author, M.M., upon reasonable request.

## Supplementary Materials

The following supporting information can be downloaded at: https://www.mdpi.com/article/10.3390/biom13030471/s1, Table S1: HCC sample list.
