# Supplementary material for: The Lysophospholipase PNPLA7 Controls Hepatic Choline and Methionine Metabolism
Source: Biomolecules. 2023 Mar 3;13(3):471. doi: 10.3390/biom13030471 (PMC10046082; doi:10.3390/biom13030471)
Supplement: Supplementary file 1 [file biomolecules-13-00471-s001.zip › biomolecules-2250946-supplementary.pdf]

Table S1.

Gender, age, and disease stages of HCC patients (related to Figure 6).

S, non-tumor: C, tumor.

	Sample mark (mRNA)	Fibrosis stage	HBV or HCV	Gender	Age
1	S2, C2	F3	nB, nC	M	65
2	S3, C3	F4	nB, nC	M	63
3	S4, C4	F1	nB, nC	M	73
4	S5, C5	F2	C	M	53
5	S6, C6	F4	C	M	63
6	S7, C7	F3	C	M	59
7	S8, C8	F4	C	M	60
8	S9, C9	F2	nB, nC	F	73
9	S10, C10	F4	C	M	63
10	S11, C11	F4	C	F	72
11	S12, C12	F1	C	M	78
12	S15, C15	F1	nB, nC	M	58
13	S16, C16	F1	nB, nC	M	69
14	S18, C18	F3	nB, nC	F	67
15	S19, C19	F4	nB, nC	M	76
16	S20, C20	F4	B, C	F	78
17	S21, C21	F4	C	F	70
18	S22, C22	F3	nB, nC	M	69
19	S23, C23	F2	C	M	84
20	S24, C24	F3	C	M	76
21	S25, C25	F2	C	M	67
22	S26, C26	F4	nB, nC	F	75
23	S27, C27	F2	B	M	57
24	S28, C28	F3	B	F	60
25	S29, C29	F4	nB, nC	M	64
26	S30, C30	F2	B	M	71
27	S31, C31	F3	C	M	83
28	S34, C34	F4	C	M	66
29	S35, C35	F1	B	M	76
30	S36, C36	F1	nB, nC	M	65
31	S37, C37	F4	nB, nC	M	75
32	S40, C40	F2	C	M	54
33	S41, C41	F4	C	F	80
34	S42, C42	F0	nB, nC	F	66
35	S43, C43	F3	B	M	66
36	S44, C44	F4	C	M	70

37	S46, C46	F4	B	M	65
38	S47, C47	F3	B	M	55
39	S48, C48	F0	nB, nC	M	64
40	S49, C49	F0	nB, nC	M	87
41	S51, C51	F4	C	F	71
42	S53, C53	F4	nB, nC	M	72
43	S54, C54	F0	nB, nC	M	78
44	S55, C55	F2	nB, nC	M	76
45	S57, C57	F3	C	M	64
46	S59, C59	F2	C	M	78
47	S60, C60	F4	C	F	66
48	S61, C61	F3	B (HBsAg+/-)	M	71
49	S62, C62	F3	nB, nC	M	71
50	S63, C63	F0	nB, nC	M	73
51	S64, C64	F0	nB, nC	M	67
52	S65, C65	F2	B	M	72
53	S66, C66	F3	nB, nC	M	71
54	S67, C67	F1	C	M	65
55	S68, C68	F4	nB, nC	M	64
56	S69, C69	F3	C	F	80
57	S70, C70	F3	nB, nC	M	68
58	S71, C71	F3	B	F	58
59	S72, C72	F4	C	F	72
60	S73, C73	F3	C	F	80
61	S74, C74	F3	nB, nC	M	71
62	S75, C75	F4	C	M	73
63	S77, C77	F1	C	F	77
64	S78, C78	F3	C	M	79
65	S79, C79	F3	nB, nC	M	68
